# Inducible deletion of microRNA activity in kidney mesenchymal cells exacerbates renal fibrosis

**DOI:** 10.1038/s41598-024-61560-y

**Published:** 2024-05-14

**Authors:** Hirofumi Sakuma, Keisuke Maruyama, Tatsuya Aonuma, Yuya Kobayashi, Taiki Hayasaka, Kohei Kano, Satoshi Kawaguchi, Kei-ichi Nakajima, Jun-ichi Kawabe, Naoyuki Hasebe, Naoki Nakagawa

**Affiliations:** 1 Division of Cardiology and Nephrology, Department of Internal Medicine, Asahikawa Medical University, Midorigaoka-Higashi 2-1-1-1, Asahikawa, Japan; 2 Department of Emergency Medicine, Asahikawa Medical University, Asahikawa, Japan; 3 Department of Biochemistry, Asahikawa Medical University, Asahikawa, Japan

**Keywords:** Dicer, miRNAs, miR-9-5p, Renal fibrosis, Renal fibrosis, miRNAs

## Abstract

MicroRNAs (miRNAs) are sequence-specific inhibitors of post-transcriptional gene expression. However, the physiological functions of these non-coding RNAs in renal interstitial mesenchymal cells remain unclear. To conclusively evaluate the role of miRNAs, we generated conditional knockout (cKO) mice with platelet-derived growth factor receptor-β (PDGFR-β)-specific inactivation of the key miRNA pathway gene Dicer. The cKO mice were subjected to unilateral ureteral ligation, and renal interstitial fibrosis was quantitatively evaluated using real-time polymerase chain reaction and immunofluorescence staining. Compared with control mice, cKO mice had exacerbated interstitial fibrosis exhibited by immunofluorescence staining and mRNA expression of PDGFR-β. A microarray analysis showed decreased expressions of miR-9-5p, miR-344g-3p, and miR-7074-3p in cKO mice compared with those in control mice, suggesting an association with the increased expression of PDGFR-β. An analysis of the signaling pathways showed that the major transcriptional changes in cKO mice were related to smooth muscle cell differentiation, regulation of DNA metabolic processes and the actin cytoskeleton, positive regulation of fibroblast proliferation and Ras protein signal transduction, and focal adhesion-PI3K/Akt/mTOR signaling pathways. Depletion of Dicer in mesenchymal cells may downregulate the signaling pathway related to miR-9-5p, miR-344g-3p, and miR-7074-3p, which can lead to the progression of chronic kidney disease. These findings highlight the possibility for future diagnostic or therapeutic developments for renal fibrosis using miR-9-5p, miR-344g-3p, and miR-7074-3p.

## Introduction

Chronic kidney disease (CKD) affects approximately 10% of the global population^1^. Importantly, delaying early-stage CKD progression provides economic benefits and prevents the development of end-stage renal disease and cardiovascular complications^2^. However, no approved therapies for CKD are currently available. The final common pathway of CKD is fibrosis caused by the activation of interstitial fibroblasts to myofibroblasts, increased secretion and accumulation of the extracellular matrix, and loss or perturbation of tissue-specific epithelial cells and other cell lineages^3–5^.

Platelet-derived growth factor receptor-β (PDGFR-β) is constitutively expressed in interstitial fibroblasts, mesangial cells, vascular smooth muscle cells, and pericytes^6^. PDGFR-β is a tyrosine-kinase receptor for platelet-derived growth factor (PDGF)-B and PDGF-D. On activation, PDGFR-β induces downstream signaling that triggers cell proliferation, migration, and differentiation^6^. Transgenic mice with PDGFR-β activation in renal mesenchymal cells had mesangioproliferative glomerulonephritis, mesangial sclerosis, and interstitial fibrosis^7^. Therefore, PDGFR-β activation is a major factor in renal fibrosis development.

MicroRNAs (miRNAs) are 18–25 nucleotide small RNA species that regulate protein translation by binding to complementary regions of mRNA. Moreover, miRNAs are increasingly recognized as important regulators of gene expression^8^. They are synthesized in the nucleus, processed by the RNase III enzyme Drosha, exported to the cytoplasm, and cleaved for subsequent activation by RNase III, also known as Dicer^8^. Therefore, Dicer is an important enzyme in miRNA biosynthesis. Recently, various mouse models with cell-specific Dicer knockout in the kidneys have been used to investigate the effects of inhibiting miRNA activity. Deletion of Dicer in podocytes results in marked proteinuria, glomerulosclerosis, and exacerbation of kidney failure^9,10^. Ablation of Dicer in the collecting duct causes tubulointerstitial fibrosis and renal failure^11^. Proximal tubule-specific Dicer knockout mice experienced increased tubular cell death, enhanced inflammation, and interstitial fibrosis in diabetic and obstructive nephropathy models^12^. During kidney development, the deletion of Dicer in Foxd1^+^ mesenchymal progenitor cells results in hypoplastic kidneys with abnormal differentiation of the nephron tubules and vasculature^13^. However, the importance of Dicer in interstitial mesenchymal cells in adult kidney diseases has not been explored. Hence, we developed a mouse model of renal interstitial mesenchymal cell-specific Dicer deletion to investigate the role of Dicer and Dicer-dependent miRNA activity in interstitial mesenchymal cells during kidney injury.

## Results

### PDGFR-β expression was upregulated with unilateral ureteral obstruction of the kidney

**Figure 1:**
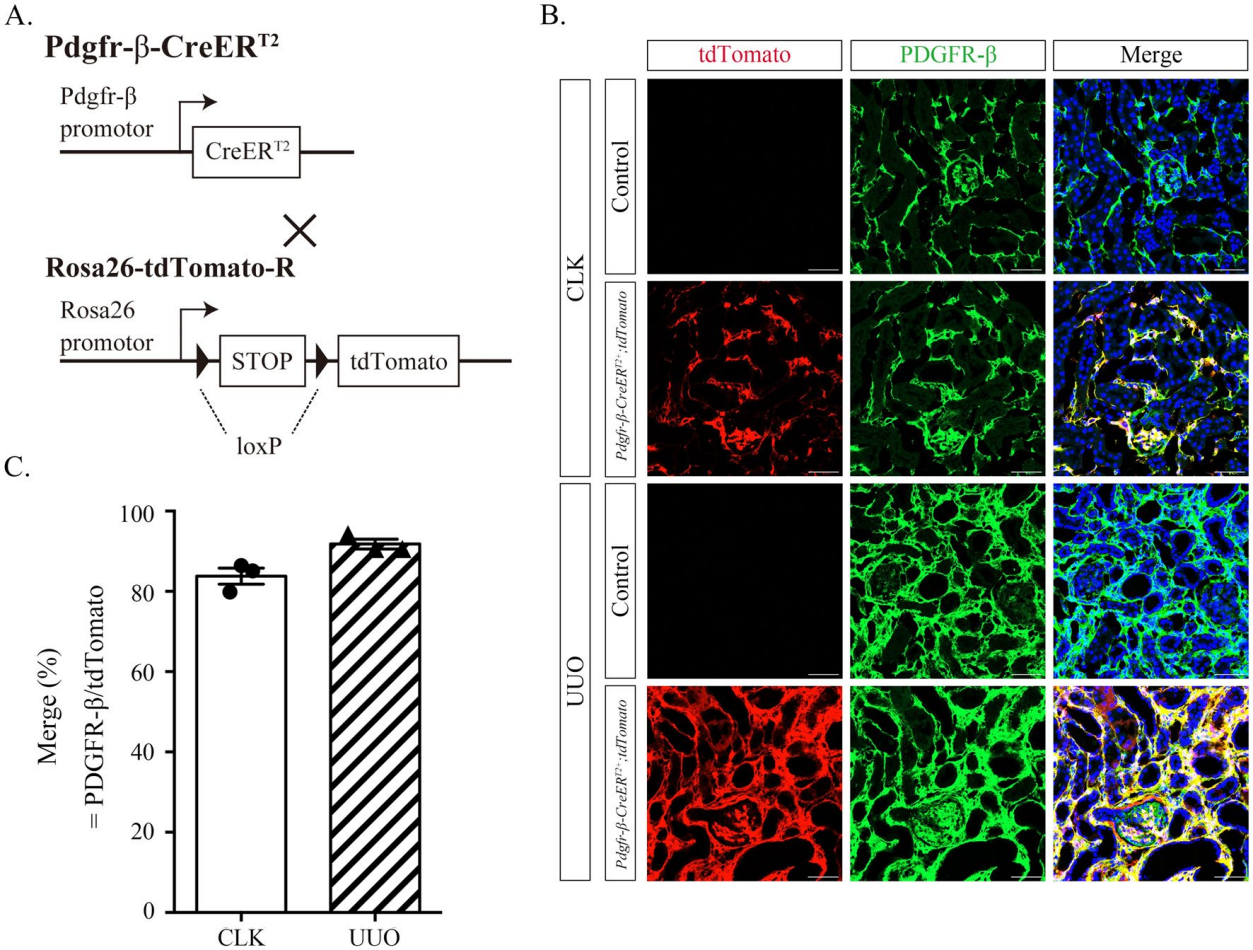
PDGFR-β expression in Pdgfr-β-CreERT2;tdTomato mice administered tamoxifen is well-localized in renal mesenchymal cells and exacerbated after UUO. (**A**) Schematic of Pdgfr-β-CreERT2 and Rosa26tdTomato mice. (**B**) Renal mesenchymal cells stained green with anti-PDGFR-β in the contralateral kidney (CLK) and UUO kidney. PDGFR-β shows colocalization of the tdTomato reporter in red. CreERT2 is absent in control mice. Scale bar = 50 µm. (**C**) Graph illustrating the proportion of tdTomato-positive mesenchymal cells expressing PDGFR-β (%PDGFR-β/tdTomato). tdTomato is effectively and specifically expressed in PDGFR-β-positive fibroblasts (n = 3).

To confirm the presence of PDGFR-β-positive cells in sham kidneys or kidneys with unilateral ureteral obstruction (UUO), *Pdgfr-β-CreER*^*T2*^ mice were crossed with *Rosa26-tdTomato* reporter mice (Fig. 1A). Then, oral tamoxifen was administered to *Pdgfr-β-CreER*^*T2*^ and *Rosa26-tdTomato* mice for 5 consecutive days to induce Cre activity. In sham kidneys, mesangial cells, vascular smooth muscle cells, pericytes, and interstitial fibroblasts were positive for PDGFR-β (Fig. 1B). PDGFR-β expression was markedly activated in kidneys with UUO compared with that in sham kidneys (Fig. 1B). Figure 1C shows the quantification of PDGFR-β-positive areas in the tdTomato-positive area, which was merged in nearly all cases.

### Inactivate Dicer in conditional knockout mice

**Figure 2:**
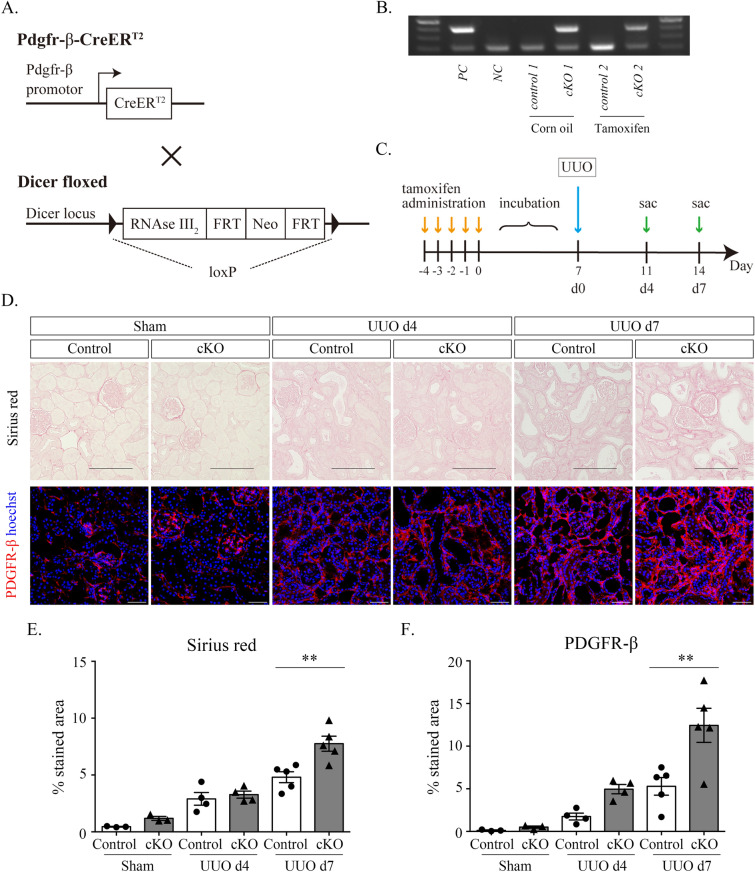
Fibrosis after UUO surgery is more exacerbated in Dicer cKO mice than in control mice. (**A**) Schematic of Pdgfr-β-CreERT2 and Dicer FL/FL mice. (**B**) Genotyping for the presence of CreERT2 using mice tail samples. The 400-bp band is detectable in Dicer cKO mice but not in control mice. (**C**) Scheme of the UUO time course. Mice are treated with tamoxifen (orally) for 8 to 12 weeks on 5 consecutive days, followed by a washout period of 7 days, subjected to UUO surgery or sham intervention, and sacrificed 4 or 7 days after surgery. (**D**–**F**). Immunohistological staining for Sirius red (**D**, upper) and PDGFR-β (**D**, lower) and histomorphometric quantification (**E**, **F**) showing that these stained areas have increased in Dicer cKO mice compared with those in control mice on UUO day 7. Data information: Scale bar = 100 µm (Sirius red) and 50 µm (PDGFR-β). Bar graphs show the mean ± standard error of the mean values of 3–5 mice per group. Statistical analysis is performed using analysis of variance with Tukey’s post hoc analysis. **p < 0.01 compared with the control at the same time point.

To inactivate the RNase III (Dicer) gene in PDGFR-β-positive cells, *Pdgfr-β-CreER*^*T2*^ mice were crossed with Dicer-floxed (*Dicer*^*FL/FL*^) mice (Fig. 2A). Exon 23 of the Dicer gene was flanked by two loxP sites in the Dicer flox allele (16, 18). This exon encodes most of the second RNase III domain; therefore, removal of the exon results in a null allele (16, 18). Polymerase chain reactions (PCRs) of genomic DNA extracted from the tails of the mice revealed that the expression of the gene coding for Cre recombinase produced a 400-bp product in the presence of Cre and no product in the absence of Cre (Fig. 2B, Supplementary Fig. S1). Additionally, the inactivation of the Dicer enzyme in the PDGFR-β-positive region was confirmed by immunostaining using Dicer antibodies (Supplementary Fig. S2A and B).

### Dicer deletion in PDGFR-β-positive cells led to increased activated PDGFR-β expression and pronounced fibrosis

**Figure 3:**
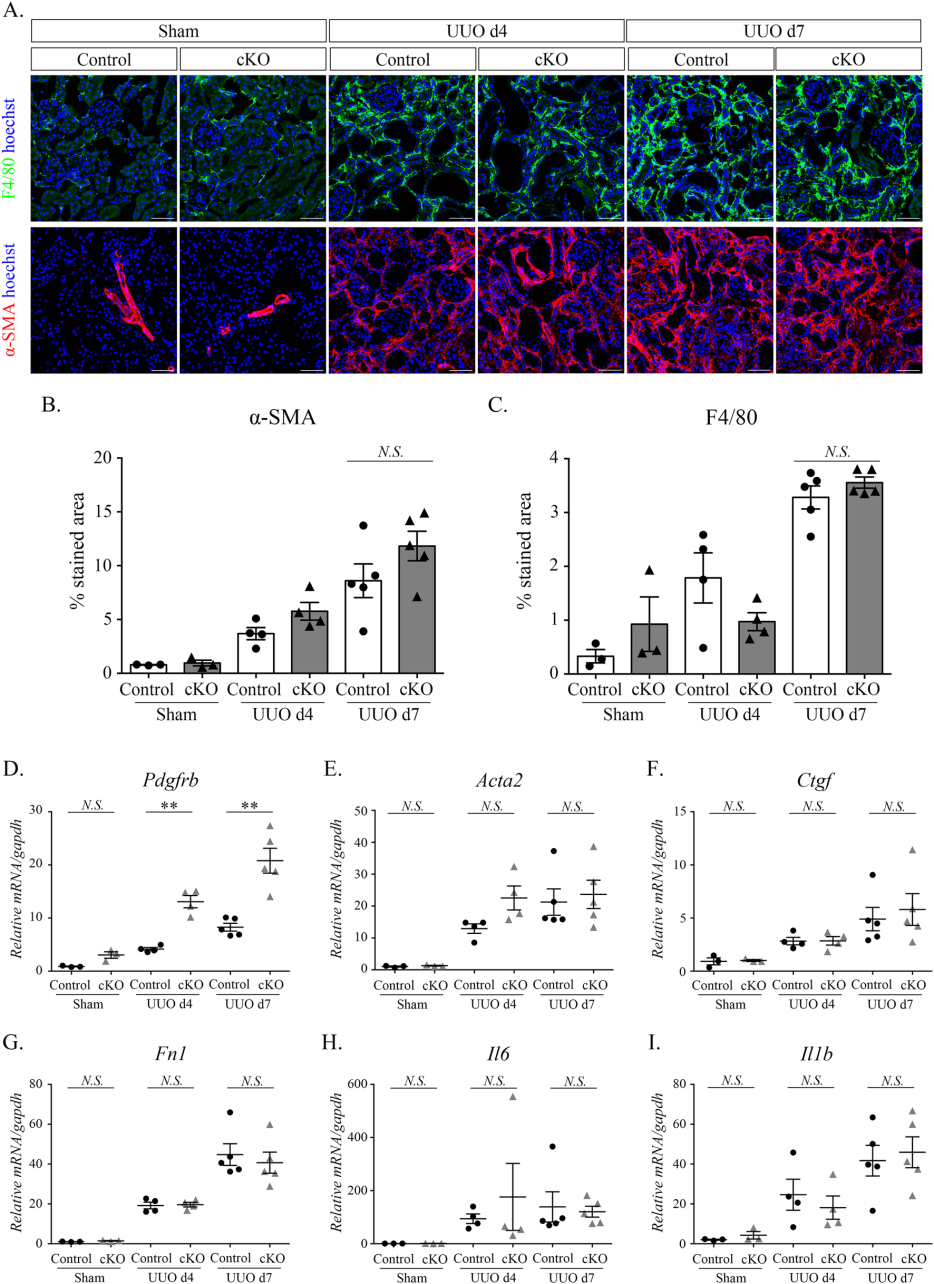
Immunohistological staining for and histomorphometric analysis of F4/80 and α-SMA show no significant differences between control and Dicer cKO mice after UUO surgery, and Dicer cKO mice have markedly increased expression of *Pdgfrb* according to the quantitative RT-PCR. (**A**–**C**). Immunohistological staining for F4/80 (**A**, upper) and α-SMA (**A**, lower) and histomorphometric quantification (**B**, **C**) showing that these stained areas are not significantly different in Dicer cKO mice at UUO day 7, compared with those in control mice. (**D**–**I**). PCR measuring markers of fibrosis (*Pdgfr*, *Ctgf*, *Acta2*, and *Fn1*) and inflammation (*Il6* and *Il1b*) of UUO mice and sham-operated mice. Data: Scale bar = 50 µm. Bar graphs show the mean ± standard error of the mean values of 3–5 mice per group. Statistical analysis is performed using analysis of variance with Tukey’s post hoc analysis. **p < 0.01, compared with the control at the same time point.

To investigate the role of Dicer in the progression of renal interstitial fibrosis, surgery was performed for UUO after tamoxifen administration, and mice were euthanized on days 4 and 7 (Fig. 2C). PDGFR-β expression markedly increased in Dicer conditional knockout (cKO) mice compared with that in control mice on days 4 and 7 after UUO (Fig. 2D,F). Additionally, renal interstitial collagen accumulation was identified using Sirius red staining. On day 7 after UUO, the percentage of collagen staining was significantly elevated in cKO mice compared with that in control mice; however, there was no significant change on day 4 after UUO (Fig. 2D,E). Although increased fibrosis was observed in cKO mice on day 7 after UUO, the α-SMA- and F4/80-stained area was not different between the groups (Fig. 3A,B and C). A quantitative reverse transcription (RT) PCR analysis demonstrated that *Pdfgrb* was upregulated in cKO mice compared with that in control mice on days 4 and 7 after UUO (Fig. 3D,E,F,G,H and I).

### Dicer deletion in PDGFR-β-positive cells led to increased PDGFR-β expression and decreased miR-9-5p

**Figure 4:**
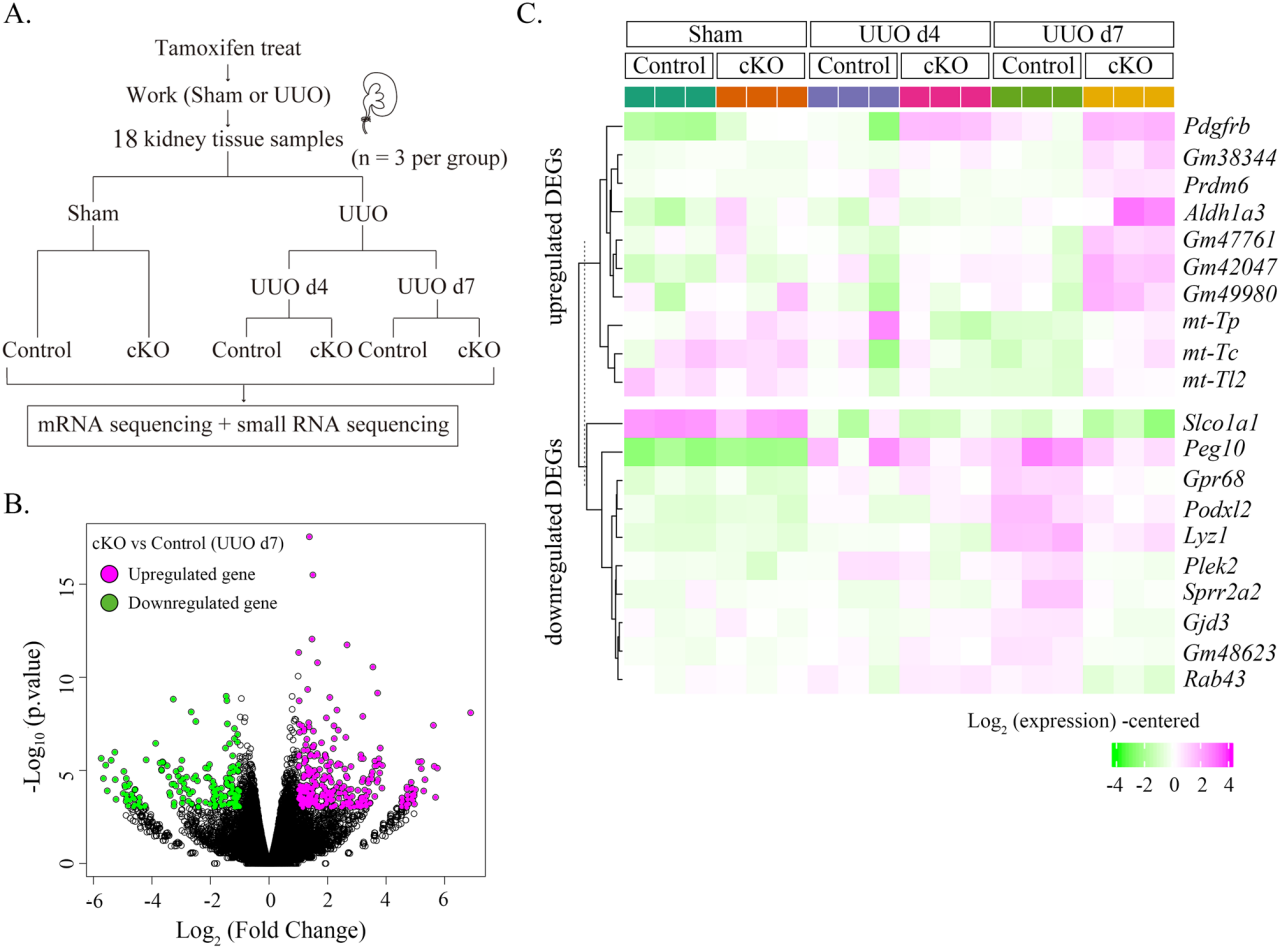
RNA sequencing showing the 20 most differentially expressed genes (DEGs) of kidneys with UUO of Dicer cKO and control mice. (**A**) Schematic of the study design and workflow for RNA sequencing. Each experimental group comprising three mice is subjected to either sham intervention or UUO followed by the collection of injured kidneys on postoperative day 4 or 7. (**B**) Volcano plot of our RNA sequence. A total of 298 upregulated and 171 downregulated DEGs are identified in Dicer cKO and control mice. Pink dots represent upregulation. Green dots represent downregulation. The X-axis depicts the log_2_ fold change in gene expression. The Y-axis depicts the –log_10_ *p*-value. (**C**) Hierarchical clustering heatmap showing the expression of the top 10 upregulated DEGs and downregulated DEGs in each group. The hierarchical clustering of genes is indicated on the left side of the heatmap, and the level of expression is indicated by color. Pink represents high gene expression. Green represents low gene expression.

To investigate factors that contribute to fibrosis exacerbation in Dicer cKO mice, sham intervention or UUO was performed in both groups, followed by the removal of kidney tissue and extraction of total RNA on postoperative day 4 or 7. Then, mice were subjected to mRNA and small RNA sequences (Fig. 4A). Principal component analysis revealed separate clustering of both groups on day 7 after UUO (Supplementary Fig. S3A). Differential expression analysis was performed using edgeR (version 3.22.3). On day 7 after UUO, Dicer cKO mice exhibited differential expression of 469 genes: 298 were upregulated and 171 were downregulated compared with those in control mice.

A volcano plot (Fig. 4B) and MAplot (Supplementary Fig. S3B) of significantly differentially expressed genes were generated (pink dots represent significantly upregulated genes and green dots represent significantly downregulated genes). On day 7 after UUO, a heatmap showed that Dicer cKO mice exhibited markedly different gene expression patterns compared with those in control mice (Supplementary Fig. S3C). The most differentially expressed genes in the two groups were highlighted during sham treatment and on days 4 and 7 after UUO (Fig. 4C). According to the analysis data, *Pdgfrb* was markedly upregulated in Dicer cKO mice compared with that in control mice on days 4 and 7 after UUO. However, there were no significant differences in *Pdgfrb* in the sham group (Fig. 4C).

**Figure 5:**
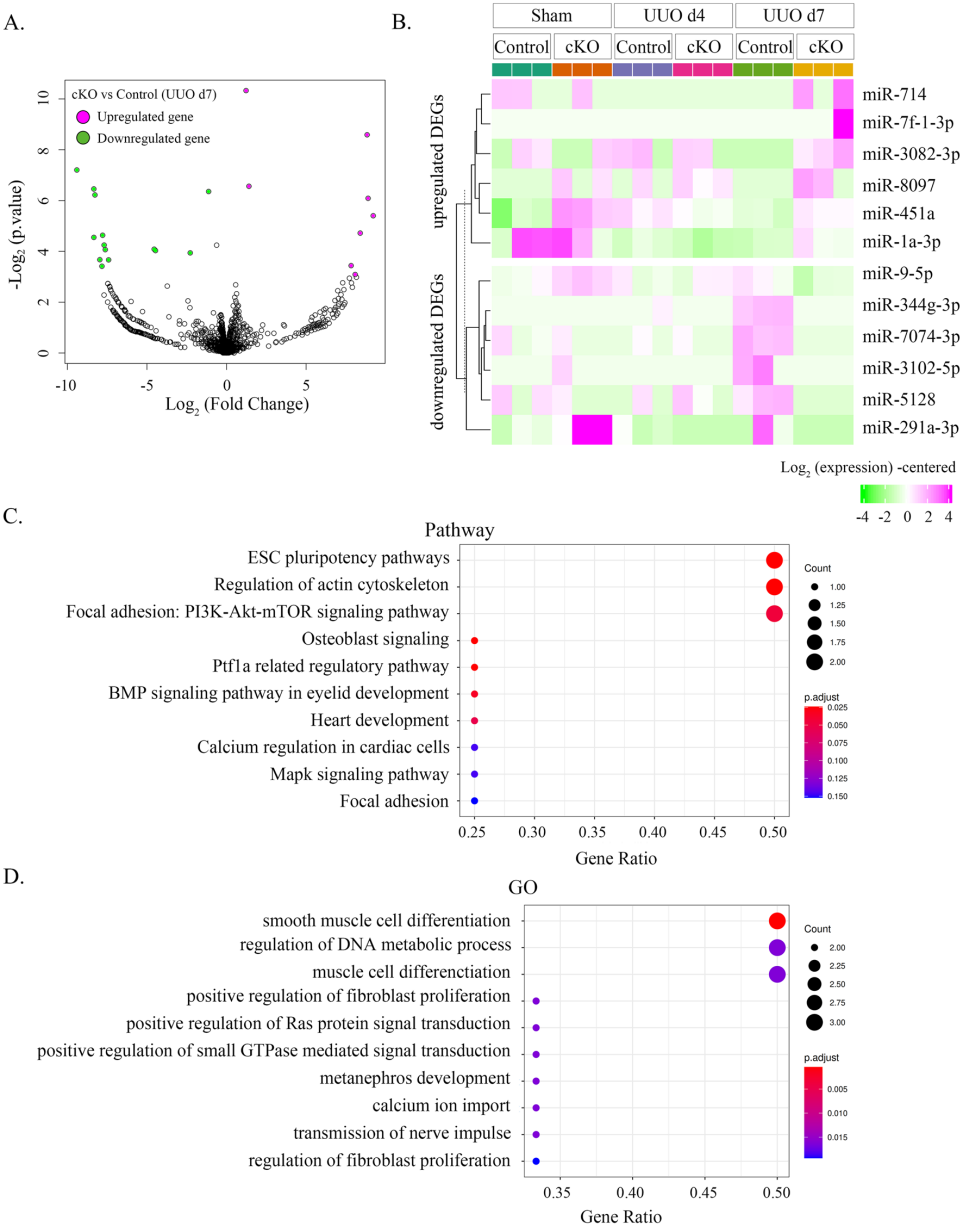
Small RNA sequencing shows differentially expressed genes (DEGs) of kidneys with UUO comparing Dicer cKO and control mice. (**A**) Volcano plot of our small RNA sequence. A total of 8 upregulated and 14 downregulated DEGs of Dicer cKO and control mice can be identified. Pink dots represent upregulation. Green dots represent downregulation. The X-axis depicts the log_2_ fold change in gene expression. The Y-axis depicts the –log_10_ *p*-value. (**B**) Hierarchical clustering heatmap showing the expression of the top six upregulated DEGs and six downregulated DEGs in each group. The hierarchical clustering of genes is indicated on the left side of the heatmap, and the level of expression is indicated by color. Pink represents high gene expression. Green represents low gene expression. (**C**, **D**) Gene Ontology and pathway analyses of control and cKO mouse kidneys 7 days after UUO.

On day 7 after UUO, Dicer cKO mice showed differential expression of 22 genes in the small RNA sequence; of these, 8 were upregulated and 14 were downregulated compared with those in control mice. The result of the volcano plot during sham treatment and on day 7 after UUO is shown in Fig. 5A. The six most differentially expressed genes in the two groups on day 7 after UUO were highlighted during sham treatment and on days 4 and 7 after UUO (Fig. 5B). According to the analysis data, miR-9-5p, 344g-3p, 3102-5p, 5128, and 7074-3p were markedly downregulated, but miR-451a, 3082-3p, and 8097 were upregulated in Dicer cKO mice compared with that in control mice on day 7 after UUO (Fig. 5B). According to the TargetScan and miRWalk databases, the potential downstream target genes of miR-9-5p, miR-344g-3p, and miR-7074-3p are included not only in PDGF family but also in the phosphoinositide-3-kinase (PI3K) family, the RAS oncogene family, mitogen-activated protein kinase (MAPK)-related protein, fibroblast growth factor (FGF) family, and mammalian target of rapamycin (mTOR)-interacting/associated protein (Supplementary Table S1).

During the integrated analysis of mRNA and miRNAs, the TargetScan database (version 7.2) was used to search for predicted target genes from downregulated miRNAs obtained by small RNA sequences. Gene sets were obtained by pairing the predicted genes with the downregulated miRNAs identified by miRNA sequencing. The Gene Ontology (GO) analysis of the obtained pair gene sets was performed using clusterProfiler (version 3.18.0). Then, Fisher’s exact probability tests of terms registered in GO (Biological Process [BP]) and terms registered in pathways were performed using clusterProfiler, and a balloon plot sorting the terms was created (Fig. 5C,D). An analysis of the signaling pathways based on pathway and GO enrichment analyses indicated that the upregulated genes were mainly involved in important pathways related to renal interstitial fibrosis, smooth muscle cell differentiation, positive regulation of fibroblast proliferation and Ras protein signal transduction, regulation of the actin cytoskeleton, focal adhesion signaling pathway, and MAPK signaling pathway (Fig. 5C,D).

### Folic acid (FA)-induced kidney injury in conditional KO mice also resulted in increased activated PDGFR-β expression and pronounced fibrosis

**Figure 6:**
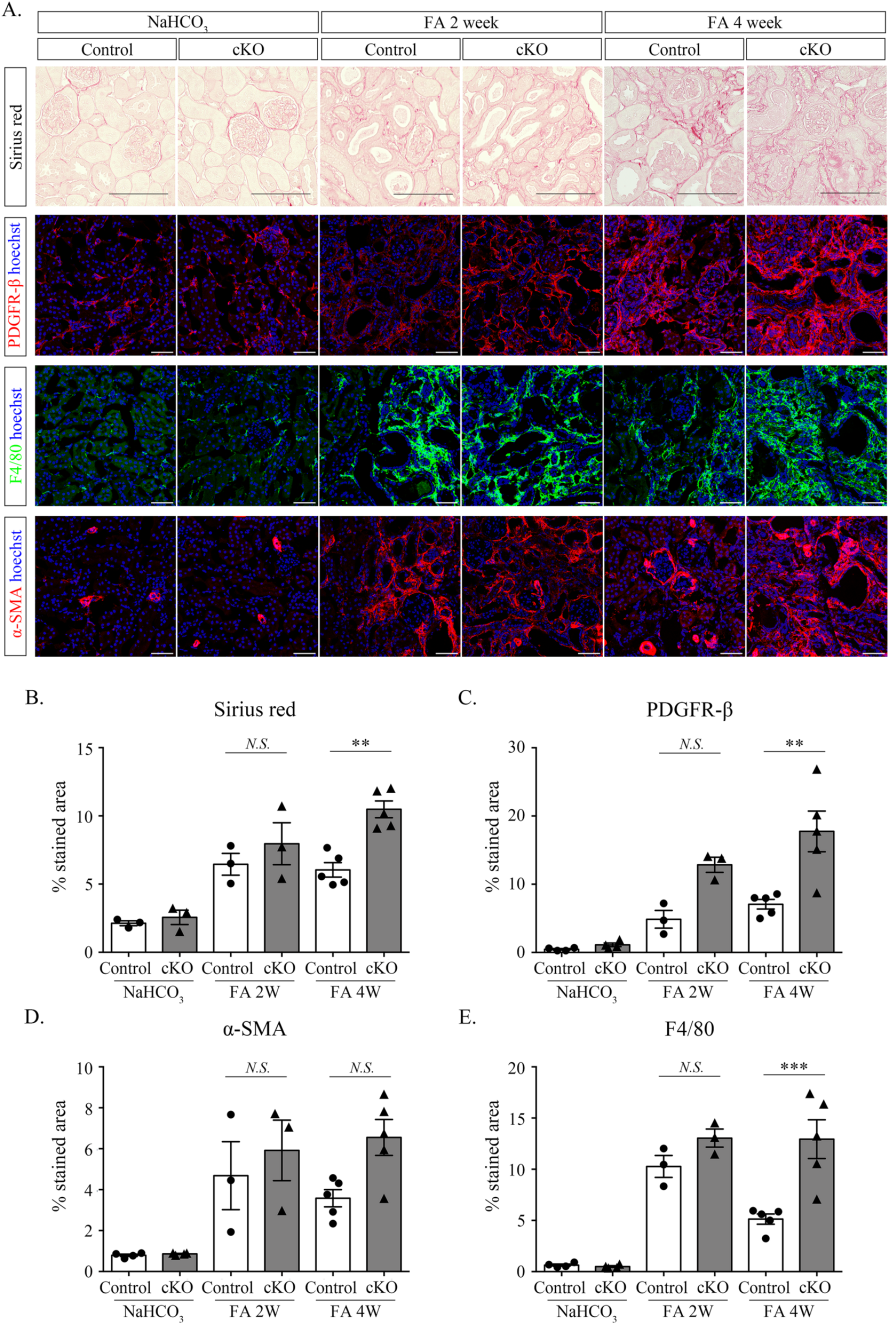
Fibrosis after injection of folic acid is more exacerbated in Dicer cKO mice than in control mice. (**A**–**E**) Immunohistological staining for Sirius red, PDGFR-β, F4/80, and α-SMA and histomorphometric quantification (**B**–**E**) showing that these stained areas increased in Dicer cKO mice compared with that in control mice on day 28. Data information: Scale bar = 100 µm (Sirius red), and 50 µm (PDGFR-β, F4/80, and α-SMA). Bar graphs show the mean ± standard error of the mean values of 3–5 mice per group. Statistical analysis is performed using analysis of variance with Tukey’s post-hoc analysis. *p < 0.05, **p < 0.01, and ***p < 0.001, compared with the control at the same time point.

Additionally, FA-induced kidney injury, as another model of renal interstitial fibrosis, was induced after tamoxifen administration, and mice were euthanized on days 14 and 28. On day 28 after FA injection, the percentage of Sirius red-positive area was significantly higher in cKO mice than in control mice (Fig. 6A,B). The expression levels of PDGFR-β and F4/80 markedly increased in Dicer cKO mice compared with those in control mice on day 28 after FA injection (Fig. 6A,C, and E); however, there was no significant change in their levels on day 14 after FA injection and in the expression of α-SMA on days 14 and 28 after FA injection (Fig. 6A and D). RT-qPCR demonstrated that *Pdfgrb*, *Acta2*, *Ctgf*, *Il6*, and *Il1b* were upregulated in cKO mice compared with that in control mice on day 28 after FA injection (Supplementary Fig. S4A–F).

### Inhibition of miR-9-5p exacerbated myofibroblast differentiation from primary renal fibroblasts

Finally, we attempted to investigate the role of miR-9-5p in renal fibrosis using primary cultured renal fibroblasts. These cells were simultaneously transfected with negative control or an miR-9-5p inhibitor, followed by treatment with TGF-β1. RT-qPCR demonstrated that *Pdgfrb*, as a target gene of miR-9-5p, and *Acta2*, as a TGF-β1-induced fibrotic gene, were significantly upregulated after miR-9-5p inhibition (Supplementary Fig. S5A and B).

## Discussion

This study revealed that renal interstitial mesenchymal cell-specific Dicer deletion led to increased activation of PDGFR-β and exacerbated renal fibrosis by decreasing miR-9-5p, miR-344g-3p, and miR-7074-3p levels. Activation of PDGFR-β was sufficient to drive renal fibrosis, and these miRNAs are potential therapeutic targets in renal fibrosis.

Interstitial fibrosis is a poor prognostic indicator of CKD and the final common pathway in progression to end-stage kidney disease, regardless of its etiology^3,4^. The recent discovery of miRNAs has facilitated important research on their potential use as therapeutic agents and targets in the clinical setting^14^. In the current study, PDGFR-β expression markedly increased according to the immunohistochemistry and quantitative PCR results of injured kidney samples obtained following PDGFR-β-positive cell-specific conditional Dicer deletion in the UUO and FA-induced kidney injury model. Furthermore, we identified a marked downregulation of miR-9-5p in kidneys with UUO in cKO mice compared with that in the kidneys of control mice during the analysis of miRNA sequences.

**Figure 7:**
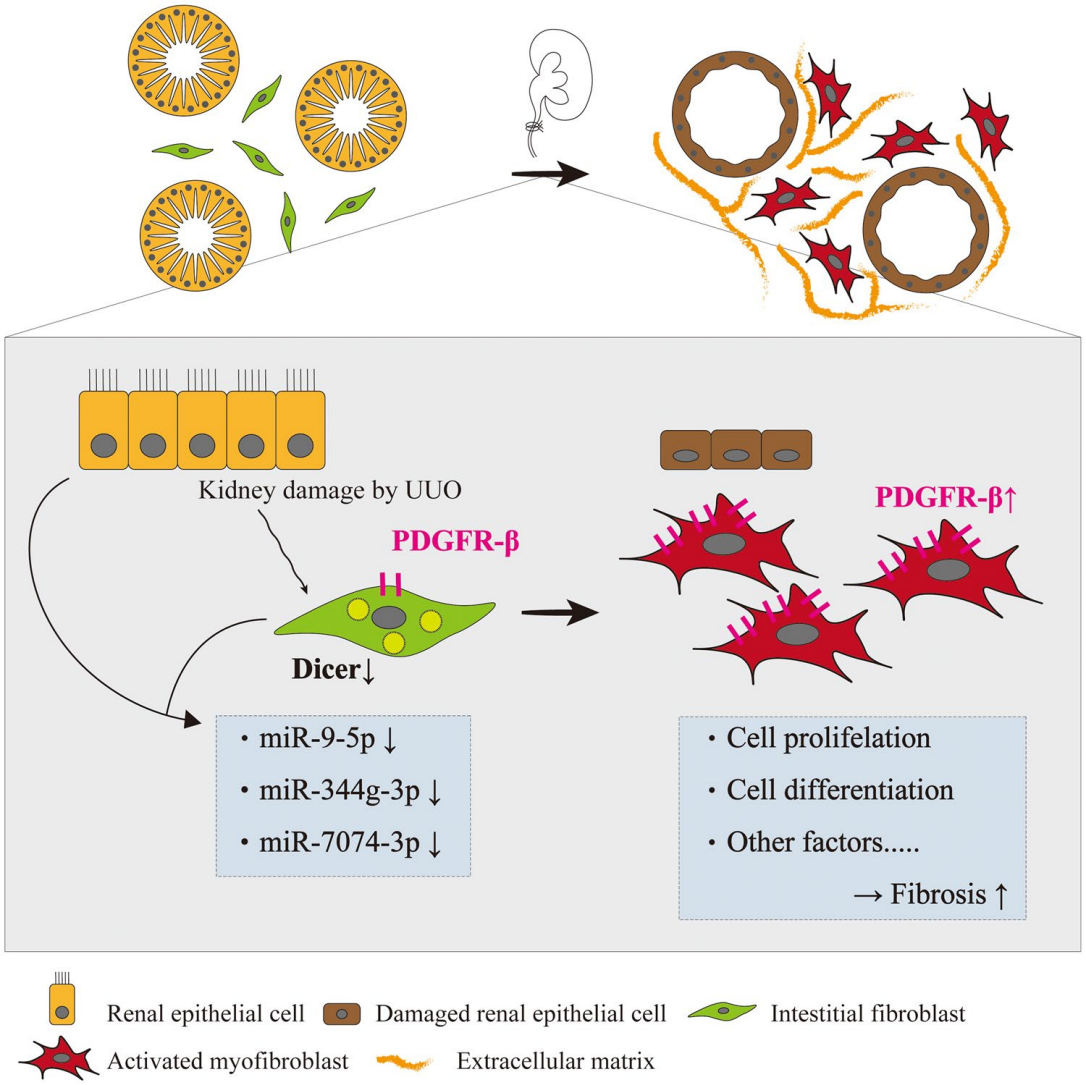
Schematic image of the proposed mechanism. Increased PDGFR-β activity resulting from reduced miR-9-5p, miR-344g-3p, and miR-7074-3p levels attributed to Dicer downregulation in renal interstitial mesenchymal cells facilitates cellular proliferation and differentiation into myofibroblasts and aggravates fibrosis.

According to the TargetScan and miRWalk databases, PDGFR-β is a target of miR-9-5p and miR-7074-3p. In a mouse model of UUO, miR-9-5p was involved in the downregulation of profibrotic factors, reduction of the number of infiltrating monocytes/macrophages, and amelioration of tubular epithelial cell damage^15^. miR-9-5p has a protective role against damage to other organs, such as the lungs^16^ and skin^17^. Therefore, we speculated that PDGFR-β overexpression in Dicer cKO mice was associated with the downregulation of miR-9-5p and that this interaction might have contributed to the exacerbation of fibrosis (Fig. 7). The potential downstream target genes of miR-9-5p, miR-344g-3p, and miR-7074-3p are included not only in the PDGF family but also in the PI3K family, the RAS oncogene family, MAPK-related protein, FGF family, and mTOR-interacting/associated protein. Future studies are needed to clarify the role of these miRNAs in renal fibrogenesis. In contrast, miR-451a, known as an anti-fibrotic miRNA^18^, was upregulated in cKO mice compared with that in control mice at day 7 after UUO. Although most miRNAs are generated through Dicer processing, miR-451 does not require this process and instead involves the catalytic activity of AGO2. It is known as a non-canonical miRNA biogenesis pathway^19^. Thus, the upregulation of miR-451a in cKO mice might be attributed to a compensatory mechanism caused by Dicer deficiency. The role of some miRs that were upregulated or downregulated with respect to their fibrosis is unclear; therefore, this is a topic for future research.

Our array data showed the upregulation of signaling pathways involved in fibrogenesis related to PDGFR-β in Dicer cKO mice after UUO. Some examples include those involved in smooth muscle cell differentiation, fibroblast proliferation, Ras protein signaling, and actin cytoskeleton formation. The array data indicated that the activation of PDGFR-β in renal interstitial cells potentiated the profibrotic pathway, resulting in exacerbated fibrosis. In another study, genetically engineered mice with PDGFR-β hyperactivation showed more exacerbated fibrosis than control mice, and it was concluded that PDGFR-β activation contributed to renal fibrosis^7^. Our findings are in line with this previous finding.

This study has some limitations. First, we analyzed RNA sequencing using total RNA from the entire kidney. Therefore, it is not completely clear whether interstitial fibroblasts or tubular epithelial cells are primarily responsible for the profibrotic effects of miR-9-5p, miR-344g-3p, and miR-7074-3p during renal fibrogenesis; however, previous studies showed that miR-9-5p significantly prevents fibrogenesis in renal tubular epithelial cells^15^ and skin fibroblasts^17^. In addition, we showed that *Pdgfrb* and *Acta2* were significantly upregulated after miR-9-5p inhibition in the cultured renal fibroblasts, suggesting that its inhibition exacerbates fibrotic effects by regulating the PDGF-PDGFR pathway. Thus, future studies are needed to clarify the effects of knockdown and overexpression of these miRNAs on renal fibrosis using kidney cell lines during an in vitro study. Second, we examined fibrosis in cKO mice by making the UUO and FA-induced kidney injury model. However, it is desirable to examine other models of CKD. Third, we could not conduct experiments to create miR-9-5p, miR-344g-3p, and miR-7074-3p knockout mice and assess their response to renal fibrosis-inducing conditions because of budgetary constraints. Further studies need to be conducted to address these issues in the future.

In conclusion, renal interstitial mesenchymal cell-specific Dicer deletion leads to increased activation of PDGFR-β by decreasing miR-9-5p, miR-344g-3p, and miR-7074-3p. PDGFR-β activation is sufficient to drive renal fibrosis, and these miRNAs are potential therapeutic targets in renal fibrosis. New insights regarding the role of Dicer and miR-9-5p in kidney injury may help identify novel candidate biomarkers and elucidate the pathophysiological processes of fibrotic kidney diseases.

## Methods

### Animals

All procedures were performed according to the protocols approved by the Animal Care and Use Committee of Asahikawa Medical University (approval number R4-058). Experiments and methods were performed in compliance with relevant regulations and the Animal Research: Reporting of In Vivo Experiments (ARRIVE) guidelines.

Transgenic mice were housed under temperatures of 20–26 °C and a 12-h light/dark cycle with free access to chow and water. The maximum caging density was five mice per aluminum cage (170 × 280 × 130 mm). Paper clean (Japan SLC, Shizuoka, Japan) was used as bedding. The general conditions of mice were checked daily. During the study, the health of all animals was maintained. *Pdgfr-β-CreER*^*T2*^, *Rosa26tdTomato*, and *Dicer*^*FL/FL*^ mice were purchased from Jackson laboratory (stock numbers 029684, 007909 and 006366, respectively). During inducible fate-tracing experiments, *Pdgfr-β-CreER*^*T2*^*;tdTomato* mice received tamoxifen (2 mg/20 g of body weight; Sigma-Aldrich, St. Louis, MO, USA) five times via oral administration, subjected to UUO surgery and sacrificed either 4 or 7 days after surgery or injection of FA and sacrificed either 14 or 28 days after injection.

Dicer cKO (*CreER*^*T2*^ + */Dicer*^*FL/FL*^) mice were obtained by crossing hemizygous *Pdgfr-β-CreER*^*T2*^ mice with homozygous Dicer-floxed (*Dicer*^*FL/FL*^) mice. The *Pdgfr-β-CreER*^*T2*^ transgenic mouse lines expressed tamoxifen-activable Cre recombinase under the control of the Pdgfr-β promoter. Dicer-floxed mice (*Dicer*^*FL/FL*^) without CreER^T2^ (*CreER*^*T2*^*-/Dicer*^*FL/FL*^) were used as control mice. Male mice were used for all experiments. Dicer cKO and control mice were administered oral tamoxifen dissolved in neutral oil for 5 consecutive days. The experiments were initiated 7 days after the last tamoxifen dose.

### Confirmation of Dicer deletion

DNA was extracted from the tails of the mice using NucleoSpin DNA RapidLyse (Takara Bio. Shiga, Japan) according to the manufacturer’s instructions and amplified with PCR using specific Dicer1 primers (forward: CCT-GAC-AGT-GAC-GGT-CCA-AAG; Dicer1 reverse: CAT-GAC-TCT-TCA-ACT-CAA-ACT). ﻿PDGFR-β-CreERT2 transgenic mice were genotyped with the PCR using specific primers (transgene forward: GAA-CTG-TCA-CCG-GGA-GGA; internal positive control forward: CAA-ATG-TTG-CTT-GTC-TGG-TG; internal positive control reverse: GTC-AGT-CGA-GTG-CAC-AGT-TT; and transgene reverse: AGG-CAA-ATT-TTG-GTG-TAC-GG). PCR products were loaded onto 1.5% agarose gels to check the Cre transgene (400 bp), its internal positive control (200 bp), the Dicer-floxed allele (420 bp), and the wild-type Dicer allele (350 bp).

### UUO model

The 8–12 week-old Dicer cKO mice (n = 9) and control mice (n = 9) underwent UUO via cauterization of the ureter, as described previously^20,21^. Briefly, male mice were anesthetized with isoflurane and placed in an abdominal position at a controlled body temperature. The left ureter was exposed through a small suprapelvic incision and ligated at two sites within the middle portion of the ureter using a 4/0 silk thread. Mice were sacrificed by exsanguination under isoflurane anesthesia either 4 or 7 days after UUO, and the kidneys were harvested.

### FA model

Eight- to twelve-week-old Dicer cKO mice (n = 11) and control mice (n = 11) were injected intraperitoneally with a single dose of FA (250 mg/kg, Sigma-Aldrich) in 0.3 M NaHCO_3_ or equal volume of vehicle (0.3 M NaHCO_3_). Mice were sacrificed by exsanguination under isoflurane anesthesia either 14 or 28 days after FA injection, and the kidneys were harvested.

### Histology, immunohistochemistry, and immunofluorescence

Following whole-body perfusion with ice-cold phosphate-buffered saline (PBS), the kidneys were removed. Paraffin-embedded kidney sections fixed in 4% paraformaldehyde were used for Sirius red staining. Interstitial fibrosis was quantified as Sirius red-positive areas, as described previously^20^. Tissue preparation for histological staining of cryosections was performed as previously described^21^. Briefly, the collected kidneys were fixed with 4% paraformaldehyde for 2 h, washed in 18% sucrose solution overnight, and embedded in FSC 22 Clear Frozen Section Compound (Leica Biosystems). For immunofluorescence, compounded samples were cut into 7-µm sections on a cryostat. The frozen sections were incubated with 0.1% Triton X-100 in PBS for 15 min at room temperature (21–25 °C) and then incubated with blocking buffer (1% bovine serum albumin in 0.1% Triton X-100 in PBS) for 30 min at room temperature. This was followed by incubation with primary antibodies overnight at 4 °C.

For antigen detection by fluorescence, primary antibodies against the following proteins were used for immunolabeling: anti-PDGFR-β (ab32570; 1:200; Abcam, Cambridge, UK), anti-α-SMA-Cy3 (1:200; clone 1A4; Sigma-Aldrich), anti-F4/80 (14-4801-82; 1:200; eBioscience, San Diego, CA, USA), and anti-Dicer (20567-1-AP; 1:100, ProteinTech, Rosemont, IL, USA). Primary antibodies were detected using fluorescence-conjugated affinity-purified secondary antibody labeling (1:1000; Thermo Fisher). Nuclei were stained with Hoechst 33,342 (H3570; Life Technologies). Images were captured using confocal fluorescence microscopes (LSM-900 [Carl Zeiss, Oberkochen, Germany] and BZ-X700 [Keyence, Osaka, Japan]).

### Quantitative RT-PCR

Total RNA was extracted using TRIzol (Thermo Fisher Scientific) and an RNeasy Mini Kit (Qiagen, Valencia, CA, USA) according to the manufacturer’s instructions^21^. To perform quantitative PCR analysis, the extracted total RNA was reverse-transcribed using the iScript Reverse Transcription Supermix (Bio-Rad, Hercules, CA, USA). Quantitative RT-PCR was performed using the TaqMan Gene Expression Master Mix (Thermo Fisher Scientific) on a LightCycler 96 (Roche). The TaqMan probe sets used for the quantitative PCR are listed in Supplementary Table S2. All values were normalized to GAPDH and plotted as fold changes versus the control.

### RNA sequencing

Libraries were sequenced using the Illumina NovaSeq 6000 platform. Sequence quality was assessed using FastQC (version 0.11.8), and low-quality sequences were removed using Trim Galore (version 0.6.4) as previously described^22^. Filtering was performed using default values, and each filtered read was mapped to the reference genome using STAR (version 2.7.0f). Additionally, counts were calculated using the gene symbols defined in GeneCode version 24. Genes with zero-count data from all samples were filtered. After filtering, all samples were normalized to align the total number of reads to 1 million counts per million.

The distance between the samples was determined using the count data after filtering. Spearman’s rank sum correlation coefficient was used with “1—correlation coefficient” as the definition of distance. Hierarchical clustering was performed using the group average method as the algorithm, and dendrograms were plotted graphically. A principal component analysis of the count data after counts per million normalization was performed, and scatter plots of PC1 to PC3 were created.

Using edgeR (version 3.22.3), *p*-values were calculated using exact probability tests for count data after Trimmed Mean of M-values normalization, and expression variation comparisons were performed. Genes with fold changes of > 2 and *p*-values of < 0.05 were defined as upregulated genes, whereas genes with fold changes of < 0.5 and *p*-values of < 0.05 were defined as downregulated genes. Map plots, volcano plots, and heatmaps were created for these genes.

GO analysis of the upregulated and downregulated genes was performed using gprofiler2 (version 0.2.1) and clusterProfiler (version 3.18.0). Using gprofiler2, Fisher’s exact probability tests were performed for terms registered in GO (BP), Molecular Function, and Cellular Component and Reactome. Then, statistical analyses were performed to determine which terms were biased toward genes with variable expression. The corrected *p*-value was obtained using the BH method, and a Manhattan plot was created with terms on the horizontal axis and the logarithm of the corrected *p*-value on the vertical axis. Next, using clusterProfiler, Fisher’s exact probability tests of terms registered in GO (BP) and WikiPathways were performed. A balloon plot was created by sorting terms in descending order of gene ratio (count data divided by the number of differentially expressed genes). GO terms with *p*-values of < 0.1 were considered indicative of a statistically significant difference^23^.

Using the TargetScan (https://www.targetscan.org/mmu_72/) and miRWalk (http://mirwalk.umm.uni-heidelberg.de/) databases, we predicted the target genes of the candidate miRNAs.

### Renal primary fibroblast culture

Mouse renal fibroblasts were isolated from 8- to 12-week-old *WT* mice following a previously reported method^20^, with minor modifications. Briefly, the kidneys were harvested, and their fibrous renal capsule and medulla were removed. The renal cortex was cut into 1 mm^3^ sections using a scalpel blade on a Petri dish with ice-cold collagenase II solution (1 mg/mL Collagenase II in DMEM/F12 with Glutamax; Gibco, Thermo Fisher Scientific, Waltham, MO, USA). The diced sections were placed in a water bath at 37 °C for 60 min with centrifugation at 150 rpm. The digested fragments were placed in collagenase II solution with ice-cold PBS/2mM EDTA and 5% BSA. The glomeruli and other tubular fragments were removed from the cell suspension by filtering them through sterilized 100-, 70-, and 40-mm sieves. The filtered solution was collected and centrifuged at 300 G for 5 min. The pellet was incubated with the Easy-lyse Erythrocyte Lysis Solution (Dako) for 5 min in ice to remove red blood cells and centrifuged at 300 G for 5 min. The pellet was resuspended with ice-cold PBS/2mM EDTA and 5% BSA and centrifuged thrice at 300 G for 5 min. After the pellet was resuspended, the primary renal fibroblasts were placed in a 2% gelatin-coated dish and incubated with DMEM/F12 with Glutamax supplemented with 20% FBS (CORNING, Corning, NY, USA) and 1% penicillin/streptomycin. After 3–5 days, renal pieces in the supernatant were gently removed, and the medium was changed. For passage, primary cultured cells were dissociated with TrypLE™ Select (Gibco Corp.,12563-011, Grand Island, NY, USA). To examine the function of miR-9-5p in primary renal fibroblasts, cells at passage 1 were used.

### Transfection of microRNA in primary cultured renal fibroblasts

Primary cultured renal fibroblasts were seeded in 6-well plates at a density of 2.0 × 10^5^/well. At 80% confluency, cells were replaced with serum-free medium (DMEM/F12 with Glutamax (Gibco, Thermo Fisher Scientific, Waltham, MO, USA) supplemented with 0.1% FBS) and then treated with 5 ng/mL recombinant human TGF-β1 for 48 h. To examine the loss of function of miR-9-5p, the cells were transfected with mirVana hsa-miR-9-5p inhibitors (20 nM; Ambion Company, USA, Cat #4464088) using the Lipofectamine RNAiMAX reagent (Thermo Fisher Scientific, Waltham, MA, USA) according to the manufacturer’s protocol. Total RNA was extracted from cultured cells using TRIzol.

### Statistical analyses

Experimental data are presented as mean ± standard error. The numbers of samples are shown in the respective figure legends. Significance was determined using an analysis of variance with Tukey’s post hoc analysis. Statistical significance was set at *p*-values of < 0.05. Data were analyzed, and graphs were constructed using the GraphPad Prism 6 for Mac OS X software package (version 6.0h; GraphPad Software, Inc., La Jolla, CA, USA).

### Supplementary Information

Supplementary Information.

## Data Availability

The datasets generated during and/or analyzed during the current study are available from the corresponding author on reasonable request.

## References

[1] GBD Chronic Kidney Disease Collaboration (2020). Global, regional, and national burden of chronic kidney disease, 1990–2017: A systematic analysis for the Global Burden of Disease Study 2017. Lancet.

[2] Kalantar-Zadeh K (2021). Chronic kidney disease. Lancet.

[3] Nakagawa N, Duffield JS (2013). Myofibroblasts in fibrotic kidneys. Curr Pathobiol Rep.

[4] Henderson NC, Rieder F, Wynn TA (2020). Fibrosis: From mechanisms to medicines. Nature.

[5] Minatoguchi S (2022). A novel renal perivascular mesenchymal cell subset gives rise to fibroblasts distinct from classic myofibroblasts. Sci. Rep..

[6] Ortiz A (2020). PDGFR-beta and kidney fibrosis. EMBO Mol. Med..

[7] Buhl EM (2020). Dysregulated mesenchymal PDGFR-beta drives kidney fibrosis. EMBO Mol. Med..

[8] Gomez IG, Nakagawa N, Duffield JS (2016). MicroRNAs as novel therapeutic targets to treat kidney injury and fibrosis. Am. J. Physiol. Renal. Physiol..

[9] Zhdanova O (2011). The inducible deletion of Drosha and microRNAs in mature podocytes results in a collapsing glomerulopathy. Kidney Int..

[10] Ho J (2008). Podocyte-specific loss of functional microRNAs leads to rapid glomerular and tubular injury. J. Am. Soc. Nephrol..

[11] Hajarnis S (2018). Suppression of microRNA activity in kidney collecting ducts induces partial loss of epithelial phenotype and renal fibrosis. J. Am. Soc. Nephrol..

[12] Ma Z (2018). Dicer deficiency in proximal tubules exacerbates renal injury and tubulointerstitial fibrosis and upregulates Smad2/3. Am. J. Physiol. Renal Physiol..

[13] Nakagawa N (2015). Dicer1 activity in the stromal compartment regulates nephron differentiation and vascular patterning during mammalian kidney organogenesis. Kidney Int..

[14] Ghafouri-Fard S (2021). Role of miRNA and lncRNAs in organ fibrosis and aging. Biomed. Pharmacother..

[15] Fierro-Fernandez M (2020). MiR-9-5p protects from kidney fibrosis by metabolic reprogramming. FASEB J..

[16] Fierro-Fernandez M (2015). miR-9-5p suppresses pro-fibrogenic transformation of fibroblasts and prevents organ fibrosis by targeting NOX4 and TGFBR2. EMBO Rep..

[17] Miguel V, Busnadiego O, Fierro-Fernandez M, Lamas S (2016). Protective role for miR-9-5p in the fibrogenic transformation of human dermal fibroblasts. Fibrogenes. Tissue Repair.

[18] Sun Y (2016). miR-451 suppresses the NF-kappaB-mediated proinflammatory molecules expression through inhibiting LMP7 in diabetic nephropathy. Mol. Cell. Endocrinol..

[19] Ha M, Kim VN (2014). Regulation of microRNA biogenesis. Nat. Rev. Mol. Cell Biol..

[20] Nakagawa N (2012). The intrinsic prostaglandin E2-EP4 system of the renal tubular epithelium limits the development of tubulointerstitial fibrosis in mice. Kidney Int..

[21] Maruyama K (2019). The antioxidant and DNA-repair enzyme apurinic/apyrimidinic endonuclease 1 limits the development of tubulointerstitial fibrosis partly by modulating the immune system. Sci. Rep..

[22] Hoshino S (2022). Elevation of the prognostic factor plasma fibrinogen reflects the immunosuppressive tumor microenvironment in esophageal squamous cell carcinoma. Ann. Surg. Oncol..

[23] Yamaguchi S (2018). Molecular and clinical features of the TP53 signature gene expression profile in early-stage breast cancer. Oncotarget.

