# Supplementary material for: Inducible deletion of microRNA activity in kidney mesenchymal cells exacerbates renal fibrosis
Source: Sci Rep. 2024 May 14;14:10963. doi: 10.1038/s41598-024-61560-y (PMC11094108; doi:10.1038/s41598-024-61560-y)
Supplement: Supplementary file 1 — Supplementary Information. [file 41598_2024_61560_MOESM1_ESM.docx]

**Inducible deletion of microRNA activity in kidney mesenchymal cells exacerbates renal fibrosis**

Hirofumi Sakuma^1^, Keisuke Maruyama^1^, Tatsuya Aonuma^1^, Taiki Hayasaka^1^, Kohei Kano^1^, Satoshi Kawaguchi^2^, Kei-ichi Nakajima^3^, Jun-ichi Kawabe^3^, Naoyuki Hasebe^1^, and Naoki Nakagawa^1^

^1^Division of Cardiology and Nephrology, Department of Internal Medicine, Asahikawa Medical University, Asahikawa, Japan

^2^Department of Emergency Medicine, Asahikawa Medical University, Asahikawa, Japan

^3^Department of Biochemistry, Asahikawa Medical University, Asahikawa, Japan

Corresponding author: Naoki Nakagawa, MD, PhD, Division of Cardiology and Nephrology, Department of Internal Medicine, Asahikawa Medical University, Midorigaoka-higashi 2-1-1-1, Asahikawa, Japan

Phone: +81-166-68-2442

Fax: +81-166-68-2449

E-mail: [naka-nao@asahikawa-med.ac.jp](mailto:naka-nao@asahikawa-med.ac.jp)

**Supplementary Figure S1.**


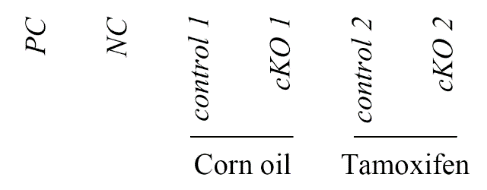

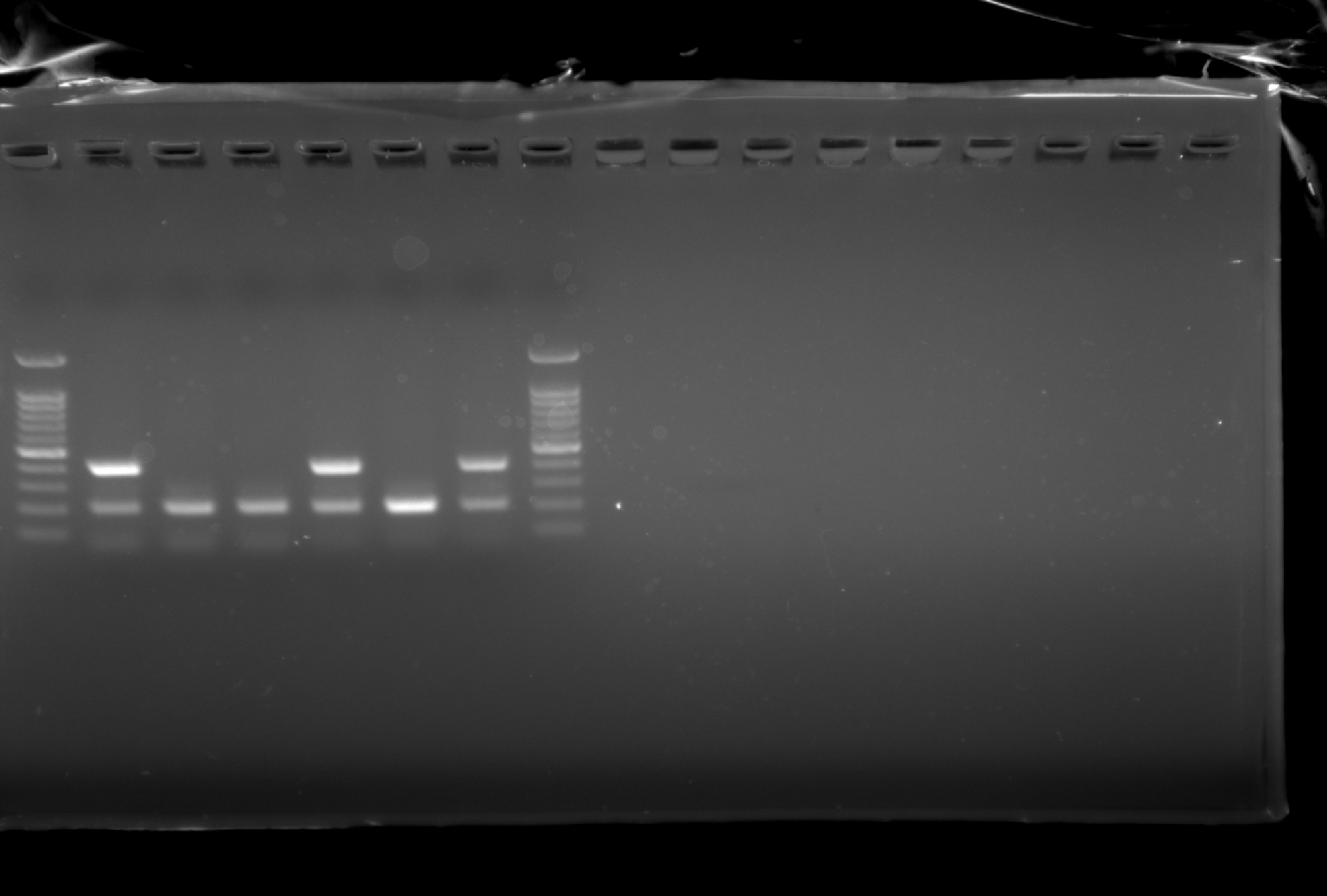


Full-length gels and blots of genotyping for the presence of CreERT2 using mice tail samples. The 400-bp band is detectable in Dicer cKO mice but not in control mice.

**Supplementary Figure S2.**


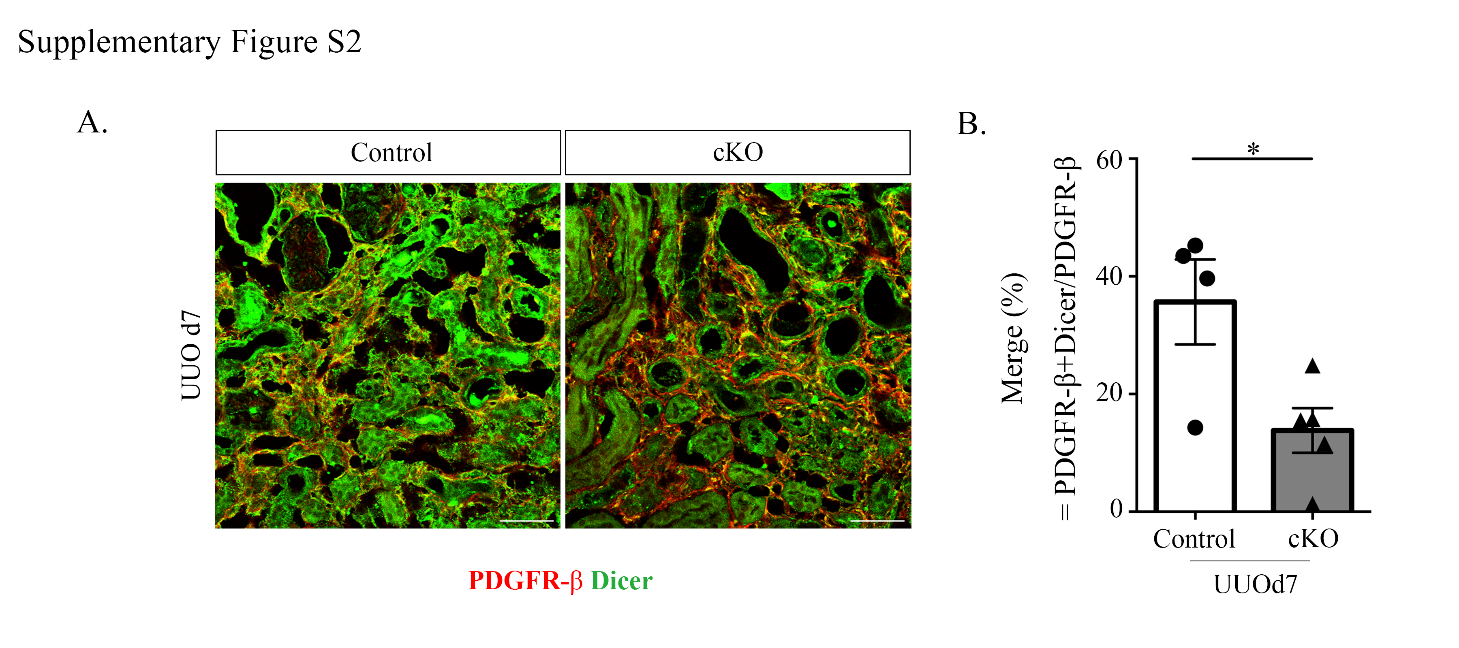


A–B. Co-immunostaining for PDGFR-β (red) and Dicer (green) in OCT kidney sections from control and cKO mice 7 d after UUO.

Data information: The bar graphs show the mean ± standard error of the mean values of 4–5 mice per group. Scale bar = 50 µm. The percentage of the merged area of PDGFR-β and Dicer was calculated using ImageJ, and statistical analysis was performed using unpaired t-test. *p < 0.05, compared with the control at the same time point.

**Supplementary Figure S3.**


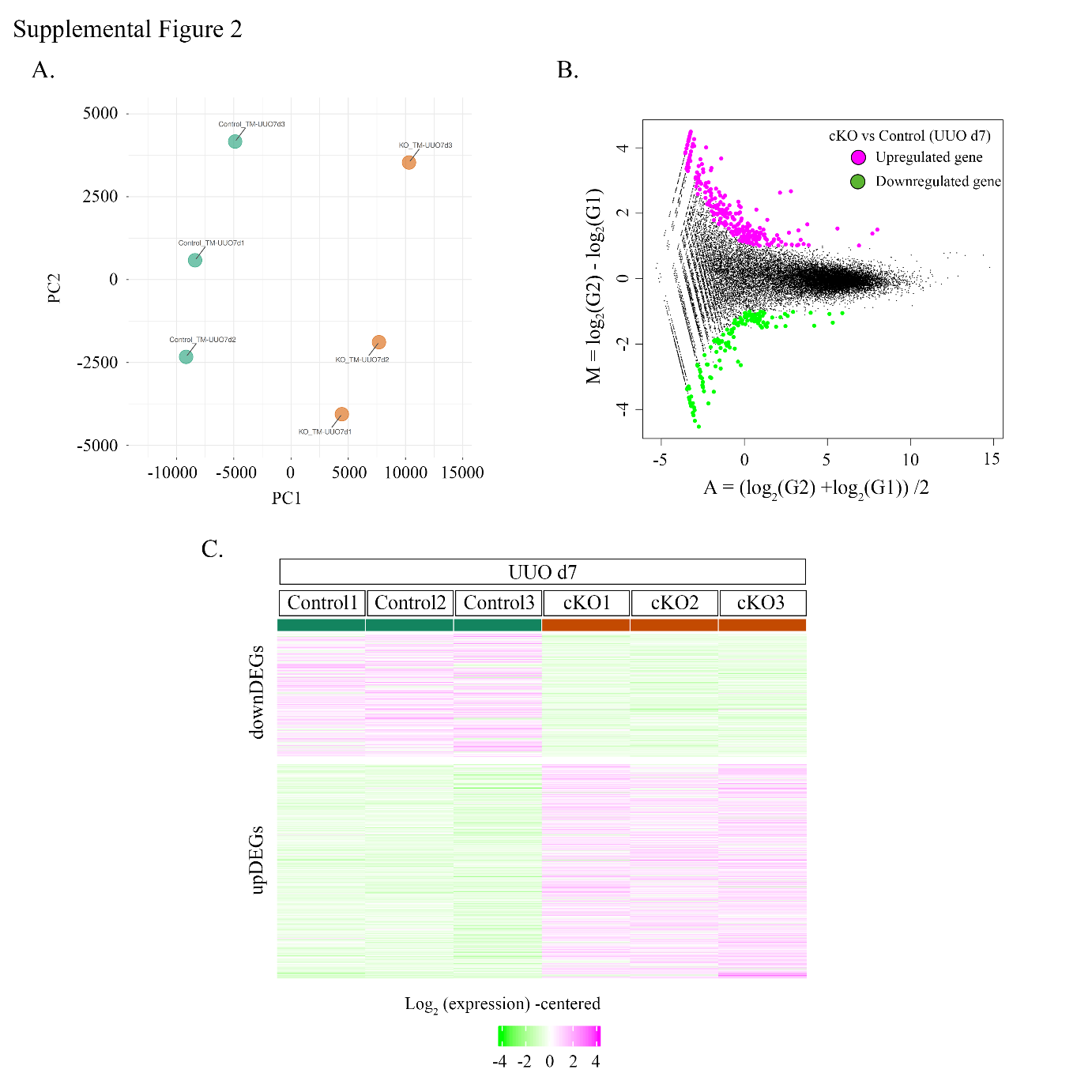


A. Principal component analysis of the UUO day 7 datasets. mRNA expression in UUO kidneys from control and Dicer cKO mice is analyzed. Green and orange dots represent control and cKO mice, respectively.

B. Log ratio (M) versus mean average (A) plot for RNA sequences derived from the kidneys on day 7 after UUO in control and cKO mice. Genes with significant differences in expression (p < 0.05) are shown in pink (upregulated) and green (downregulated).

C. Heatmap of DEG of kidneys at day 7 after UUO between control and cKO mice (n = 3 mice per group). Expression levels are indicated in color. Pink represents highly expressed genes, and green represents genes with low expression.

**Supplementary Figure S4.**


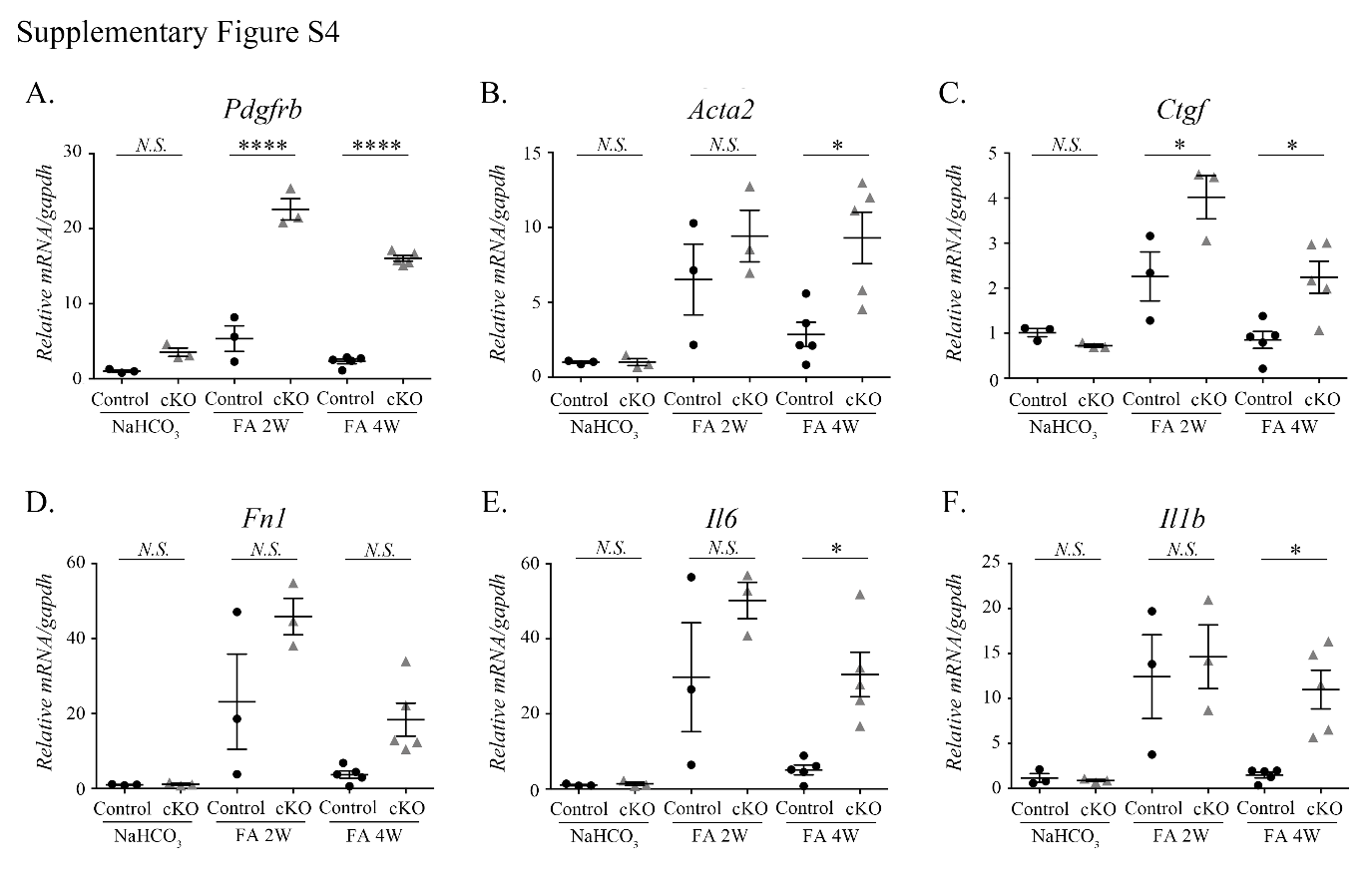


A–F. PCR measuring markers of fibrosis (*Pdgfrb, Acta2, Ctgf, and Fn1*) and inflammation (*Il6 and Il1b*), comparing folic acid-treated mice and control mice.

Data: Bar graphs show the mean ± standard error of the mean values of 3–5 mice per group. Statistical analysis was performed using analysis of variance with Tukey’s post-hoc analysis. *p < 0.05, and ****p < 0.0001, compared with control at the same time point.

**Supplementary Figure S5.**


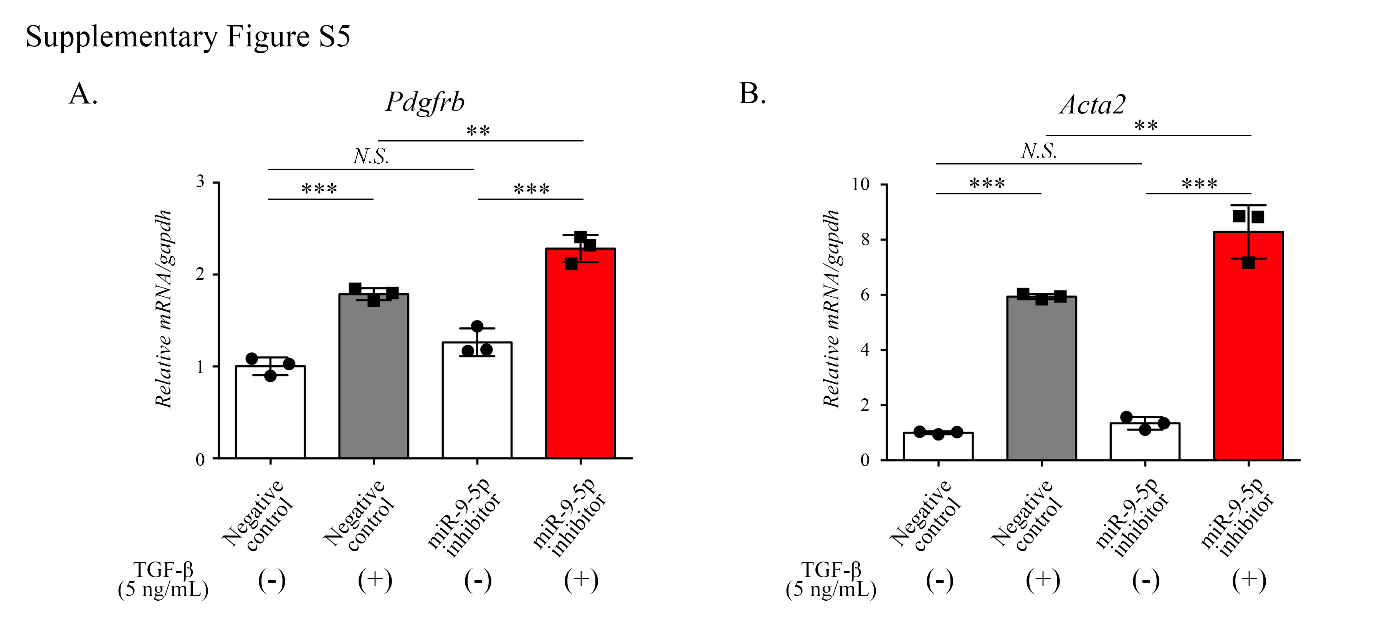


A–B. Primary cultured renal fibroblasts were transfected with negative control or miR-9-5p inhibitor and treated with TGF-β1 (5 ng/mL) for 48 hours, the mRNA levels of *Pdgfrb* and *Acta2* were measured by PCR.

Data: Bar graphs show the mean ± standard error of the mean values of three wells per group. Statistical analysis is performed using analysis of variance with Tukey’s post-hoc analysis. **p < 0.01, and ***p < 0.001, compared with control at the same time point.

**Supplementary Table S1. Abbreviated list of candidate target genes for each miRNAs**

| **miRNA** | **Gene Description** | **Targets** |
| --- | --- | --- |
| mmu-miR-9-5p | fibroblast growth factor (FGF) family | Fgf4, Fgf5, Fgf6, Fgf7, Fgf9, Fgf10, Fgf12, Fgf13, Fgf14, Fgf17, Fgf18, Fgf22 |
|  | mitogen-activated protein (MAP) kinase family | Mapk4, Mapk9, Mapk10, Mapk11, Map2k3, Map2k6, Map2k7, Map3k1, Map3k2, Map3k3, Map4k2, Mapkbp1 |
|  | late endosomal/lysosomal adaptor, MAPK, and MTOR activator 3 | Lamtor3 |
|  | MTOR-associated protein, LST8 homolog | Mlst8 |
|  | platelet-derived growth factor family | Pdgfc, Pdgfrb |
|  | phosphoinositide 3-kinase (PI3K) family | Pik3ap1, Pik3c2a, Pik3c2b, Pik3c3, Pik3cb, Pik3cd, Pik3cg, Pik3r3, Pik3r5, Pik3r6 |
|  | RAS oncogene family | Rab11b, Rab12, Rab15, Rab21, Rab23, Rab27a, Rab29, Rab2b, Rab30, Rab34, Rab37, Rab38, Rab39, Rab40b, Rab43, Rab44, Rab5b, Rab9, Rab9b, Rap1b, Rap2a, Rap2c |
| mmu-miR-344g-3p | fibroblast growth factor (FGF) family | Fgf1, Fgf6, Fgfr1op2, Fgfr3, Fgfr11 |
|  | mitogen-activated protein (MAP) kinase family | Mapk8, Mapk9, Map2k2, Map2k6, Map3k5, Map3k9, Map3k11, Map3k12, Map3k13, Map4k2, Map4k3 |
|  | late endosomal/lysosomal adaptor, MAPK, and MTOR activator 3 | Lamtor3 |
|  | MTOR-associated protein, LST8 homolog | Mlst8 |
|  | platelet-derived growth factor family | Pdgfa, Pdgfb |
|  | phosphoinositide 3-kinase (PI3K) family | Pik3ap1, Pik3cb, Pik3cd, Pik3r1, Pik3r2, Pik3r5, Pik3r6 |
|  | RAS oncogene family | Rab1b, Rab6b, Rab7, Rab8b, Rab11b, Rab18, Rab22a, Rab23, Rab27b, Rab28, Rab29, Rab31, Rab39, Rab43 |
| mmu-miR-7074-3p | fibroblast growth factor (FGF) family | Fgf1, Fgf4, Fgf12, Fgf16, Fgfr1, Fgfr2, Fgfr4 |
|  | mitogen-activated protein (MAP) kinase family | Mapkap1, Mapk3, Mapk8ip3, Mapk10, Mapk13, Map2k3, Map2k6, Map3k2, Map3k5, Map3k8, Map3k9, Map4k4 |
|  | late endosomal/lysosomal adaptor, MAPK, and MTOR activator 3 | Lamtor3 |
|  | Regulatory-associated protein of MTOR complex 1 | Rptor |
|  | platelet-derived growth factor family | Pdgfrb |
|  | phosphoinositide 3-kinase (PI3K) family | Pik3c2g, Pik3c3, Pik3r1, Pik3r3 |
|  | RAS oncogene family | Rab2b, Rab3c, Rab3d, Rab5b, Rab8a, Rab9, Rab9b, Rab11b, Rab19, Rab27a, Rab29, Rab35 |

**Supplementary Table S2. The TaqMan probe sets used for qPCR**

| Gene Name | TaqMan Probe | Species |
| --- | --- | --- |
| *Acta2* | Mm00725412_s1 | Mouse |
| *Ctgf* | Mm01192933_g1 | Mouse |
| *Dicer1* | Mm00521730_m1 | Mouse |
| *Fn1* | Mm01256744_m1 | Mouse |
| *Gapdh* | Mm99999915_g1 | Mouse |
| *IL1b* | Mm00434228_m1 | Mouse |
| *IL6* | Mm00446190_m1 | Mouse |
| *Pdgfrb* | Mm00435546_m1 | Mouse |
